# Transcriptomic Profiling of Tomato Leaves Identifies Novel Transcription Factors Responding to Dehydration Stress

**DOI:** 10.3390/ijms24119725

**Published:** 2023-06-03

**Authors:** Shuchao Dong, Jiayi Ling, Liuxia Song, Liping Zhao, Yinlei Wang, Tongmin Zhao

**Affiliations:** 1 Institute of Vegetable Crop, Jiangsu Academy of Agricultural Sciences, Nanjing 210014, China; 20221007@jaas.ac.cn (S.D.); mz120211352@stu.yzu.edu.cn (J.L.); songliuxia@jaas.ac.cn (L.S.); zhaoliping_mail@126.com (L.Z.); yinleiwang@163.com (Y.W.); 2 Laboratory for Genetic Improvement of High Efficiency Horticultural Crops in Jiangsu Province, Nanjing 210014, China; 3 College of Horticulture and Landscape Architecture, Yangzhou University, Yangzhou 225100, China

**Keywords:** tomato, dehydration response, drought tolerance, RNA-Seq, transcriptome, transcription factor

## Abstract

Drought is among the most challenging environmental restrictions to tomatoes (Solanum lycopersi-cum), which causes dehydration of the tissues and results in massive loss of yield. Breeding for dehydration-tolerant tomatoes is a pressing issue as a result of global climate change that leads to increased duration and frequency of droughts. However, the key genes involved in dehydration response and tolerance in tomato are not widely known, and genes that can be targeted for dehydration-tolerant tomato breeding remains to be discovered. Here, we compared phenotypes and transcriptomic profiles of tomato leaves between control and dehydration conditions. We show that dehydration decreased the relative water content of tomato leaves after 2 h of dehydration treatment; however, it promoted the malondialdehyde (MDA) content and ion leakage ratio after 4 h and 12 h of dehydration, respectively. Moreover, dehydration stress triggered oxidative stress as we detected significant increases in H_2_O_2_ and O^2−^ levels. Simultaneously, dehydration enhanced the activities of antioxidant enzymes including peroxidase (POD), superoxide dismutase (SOD), catalase (CAT), and phenylalanine ammonia-lyase (PAL). Genome-wide RNA sequencing of tomato leaves treated with or without dehydration (control) identified 8116 and 5670 differentially expressed genes (DEGs) after 2 h and 4 h of dehydration, respectively. These DEGs included genes involved in translation, photosynthesis, stress response, and cytoplasmic translation. We then focused specifically on DEGs annotated as transcription factors (TFs). RNA-seq analysis identified 742 TFs as DEGs by comparing samples dehydrated for 2 h with 0 h control, while among all the DEGs detected after 4 h of dehydration, only 499 of them were TFs. Furthermore, we performed real-time quantitative PCR analyses and validated expression patterns of 31 differentially expressed TFs of NAC, AP2/ERF, MYB, bHLH, bZIP, WRKY, and HB families. In addition, the transcriptomic data revealed that expression levels of six drought-responsive marker genes were upregulated by de-hydration treatment. Collectively, our findings not only provide a solid foundation for further functional characterization of dehydration-responsive TFs in tomatoes but may also benefit the improvement of dehydration/drought tolerance in tomatoes in the future.

## 1. Introduction

Drought stress is a major concern in agriculture, as it affects plant growth and crop production. It typically occurs when the irrigation water is limited in soil or dehydration is caused by transpiration or evaporation under dry atmospheric conditions [1]. Despite technological advances in water supply, fresh water is still insufficient given its extensive use in agriculture. Globally, drought is among the top causes of agricultural production losses and frequently coincides with elevated temperatures and radiation [2,3]. Droughts occurred 293 times worldwide and caused total damage costing USD 107.2 billion between 2001 and 2018. China suffered 20 droughts during this period, which was the most frequent in contrast to other countries and regions (accessed on 21 March 2020, https://www.unwater.org/). Drought induces massive changes in plants at the organ, tissue, cell, and molecular levels [4,5]; for instance, dehydration of plant organs, decreased chlorophyll content, and photosynthesis efficiency [6,7,8]. Subsequently, dehydration induces peroxidation of lipids resulting in increased electrolyte leakage due to membrane damage, as well as triggering excessive accumulation of ROS (reactive oxygen species) [9,10,11]. Drought leads to dehydration effects on plants. Moreover, the drought tolerance of plants is a complex phenotype, which includes an important component: dehydration tolerance [12].

Tomato is a widely consumed horticultural crop and one of the leading fruit crops worldwide. Exposure to dehydration is one of the major sources of stress for tomato plants; therefore, it is of great importance and necessity to advance the understanding of dehydration response in tomatoes in order to improve their ability to grow with less water [13]. In addition, tomato is also a valuable research model due to the genetic, genomic, and molecular tools developed by the research community over the years [14]. Research on dehydration response has been conducted over recent decades to illustrate how tomato plants respond to dehydration stress including physiological and biochemical processes and strategies for controlling water status [15]. An additional crucial aspect of dehydration that has been addressed by many studies is its effects on the agronomic traits of tomatoes [16,17]. For instance, the total tomato production in Xinjiang province of China decreased by 51.63% in 2012 due to long exposure to dehydration stress. Therefore, the enhancement of dehydration tolerance remains one of the major breeding goals in tomatoes.

Many factors have been shown to impact dehydration tolerance in tomato: microRNAs [18], TFs [19], osmotic regulators [12], phytohormones [20], stress proteins [21], and metabolic genes [22,23]. TFs perform their biological functions by regulating the transcription of target genes and play key roles in regulating plant growth, development, and stress response [24,25]. Studies have shown that plant TFs can regulate plant dehydration resistance by simultaneously controlling multiple genes via transcriptional cascades in the dehydration response pathway; for instance, genes related to ROS, ABA (abscisic acid), and osmoregulation [20,26,27]. Thus, TFs can be promising targets for the breeding of dehydration/drought-tolerant crops. 

The plant TF database (http://planttfdb.gao-lab.org/index.php (accessed on 29 May 2023)) shows that the tomato genome contains a total number of 1845 transcription factors, which are clustered into 58 families based on their protein sequences and structural features [28,29,30,31]. Only a few TFs of different families have been functionally characterized as regulators controlling dehydration responses in tomatoes. For example, SlJUB1, a member of the tomato NAC family, was identified as an important player in drought and dehydration response. The expression of SlJUB1 was strongly induced by dehydration and overexpressing SlJUB1 enhanced drought tolerance in tomatoes [10]. Zhu et al. found that silencing the expression of bZIP gene SlbZIP1 significantly decreased the survival rate of transgenic tomatoes after dehydration treatment [32]. A WRKY gene, SlWRKY81, was reported to improve the dehydration resistance of tomatoes by accelerating stomatal closure, increasing the accumulation of proline, and reducing levels of H_2_O_2_ and MDA (malondialdehyde) [33,34]. These indeed confirmed that tomato TFs play important roles in dehydration response. Hence, these TFs are profitable genetic resources for improving dehydration tolerance in tomatoes. However, TFs that regulate dehydration response or dehydration tolerance remain to be identified and characterized. 

We previously demonstrated the physiological effects of drought stress on tomatoes [2]. However, the molecular mechanism and genes involved in drought responses remain to be discovered. In this study, we detached tomato leaves for dehydration experiments to mimic drought stress. Our results revealed similar effects of dehydration stress on tomato leaves to drought stress, suggesting an overlap in the responsive pathways triggered by drought and dehydration stress.

Although several transcriptome analyses have been reported to identify genes responding to dehydration stress in tomatoes [15,35], they only presented DEGs (differentially expressed genes) from the genome-wide level instead of specific groups of genes. Here, we performed genome-wide RNA sequencing of tomato leaves treated with or without dehydration, and specifically focused on differentially expressed TFs. The RNA-seq analysis identified numerous DEGs annotated as TFs at both time points (2 h of dehydration vs. 0 h control and 4 h of dehydration vs. 0 h control) belonging to NAC, AP2/ERF, MYB, bHLH, bZIP, WRKY, HB, and other families. Out of these differentially expressed TFs, we validated the expression patterns of 31 TFs of different families by real-time quantitative PCR analyses. Notably, several drought-responsive marker genes showed increased expression levels after dehydration. In summary, our work provides novel insights into the regulatory complexity of the transcriptional control of dehydration response in tomatoes and suggests new entry points for breeding dehydration/drought-tolerant tomatoes.

## 2. Results

### 2.1. Dehydration Stress Has a Major Impact on Tomato Leaves

**Figure 1:**
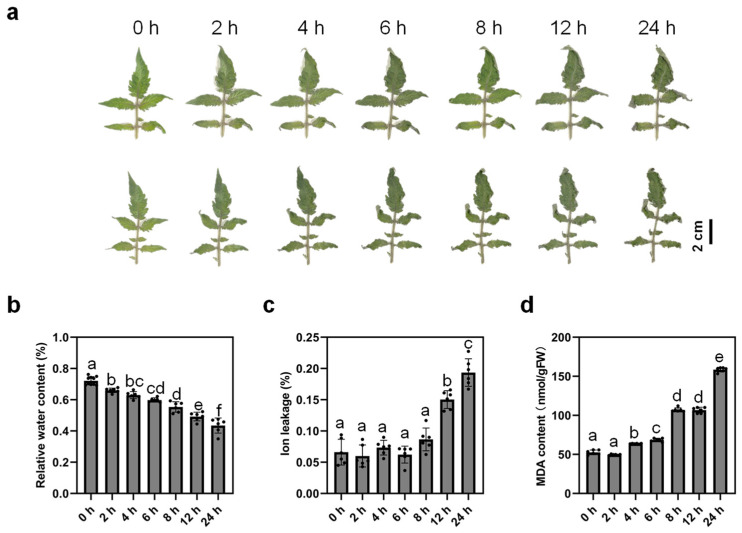
Detached leaves of MicroTom (MT) in response to drought. (**a**) Phenotypes of MT leaves under dehydration conditions. The 4th leaf of one-month-old MT plants was subjected to dehydration treatment and photos were taken during dehydration treatment (0, 2, 4, 6, 8, 12, and 24 h). Relative water content (**b**), ion leakage (**c**), and MDA (malondialdehyde) content (**d**) of MT leaves after 0, 2, 4, 6, 8, 12, and 24 h of dehydration treatment. Data represent means ± SD (*n* = 6–10). In (**b**–**d**), letters indicate significant differences between means (*p* < 0.05; one-way ANOVA).

In order to investigate the dehydration response in tomatoes, tomato leaves were detached and placed on filter papers in a growth chamber for desiccation. The tomato leaves exhibited a swift response to dehydration treatment. The manifestation of wilting characteristic was observed within 2 h of dehydration, and its intensity increased progressively with the continuation of the treatment. To track the physiological changes caused by dehydration stress in tomato leaves, relative water content, ion leakage, and MDA content were measured from 0 h to 24 h during dehydration treatment. As shown in Figure 1b, the relative content of tomato leaves dropped significantly after 2 h of dehydration in contrast to 0 h and decreased gradually over the duration of the treatment (Figure 1b), which was consistent with the phenotype observed in Figure 1a. The ion leakage rate was significantly increased at two time points, 12 h and 24 h after dehydration compared with 0 h (Figure 1c), indicating that membrane damage occurred after 12 h dehydration. In addition, the content of MDA quantified after 4 h, 6 h, 8 h, 12 h, and 24 h of dehydration was significantly higher than 0 h (Figure 1d), which suggests the enhancement of membrane lipid peroxidation started after 4 h of dehydration. Previous studies showed that drought stress affected chlorophyll accumulation in tomato leaves [8]. However, the chlorophyll content remained stable through the time window analyzed in this study (Figure S1). Taken together, these results showed that dehydration stress exerted strong phenotypical and physiological impacts on tomato leaves.

### 2.2. Dehydration Induces Oxidative Stress Responses

**Figure 2:**
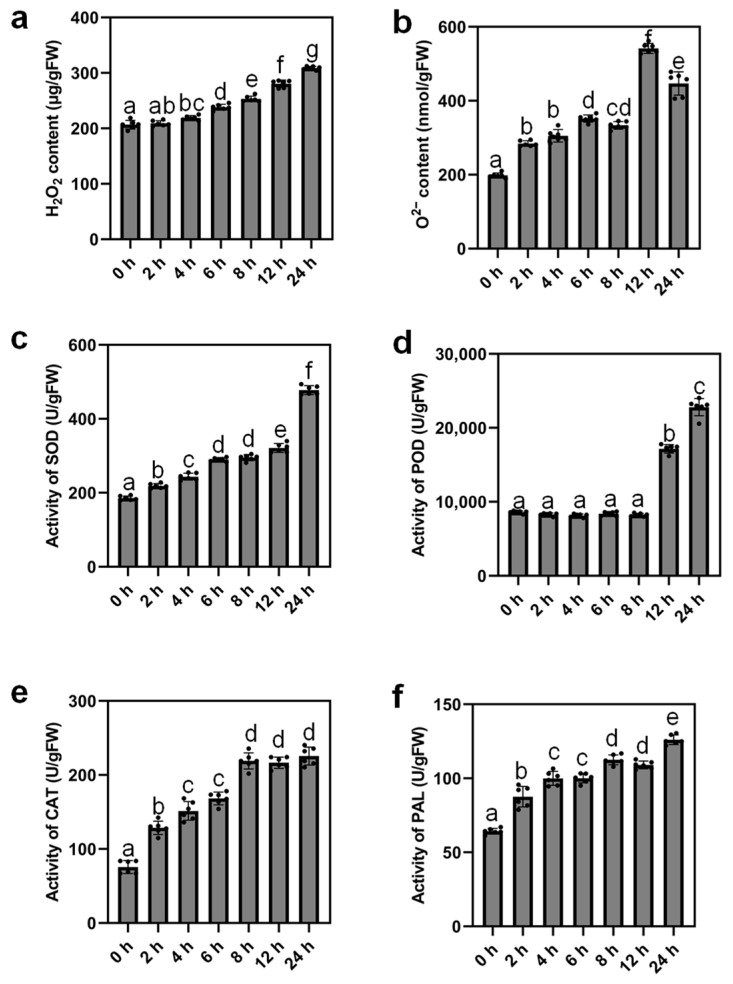
Oxidative stress response induced by drought in MT leaves. H_2_O_2_ content (**a**), O^2−^ content (**b**), enzymatic activities of superoxide dismutase (SOD) (**c**), peroxidase (POD) (**d**), catalase (CAT)CAT (**e**), and phenylalanine ammonia-lyase (PAL) (**f**) were determined in detached leaves of MT after 0, 2, 4, 6, 8, 12, and 24 h of dehydration treatment. Values reflect means ± SD of six biological replicates and letters denote significant differences between means (*p* < 0.05; one-way ANOVA).

The response to water deprivation in tomatoes is a dynamic and complex process. It has been shown that oxidative stress and responses could be triggered by drought [2,36]. To verify whether dehydration treatment also induces oxidative stress in tomato leaves, we analyzed the content of H_2_O_2_ and O^2−^. Our results indeed revealed the emergence of oxidative stress when leaves were dehydrated. A significant increase in the H_2_O_2_ content was observed after 4 h of dehydration and a gradual increase was also observed at the latter time points we analyzed (Figure 2a). Whereas, the O^2−^ content started to increase significantly after dehydration for 2 h and peaked after 12 h of dehydration treatment (Figure 2b). We also detected enhanced activities of antioxidant enzymes (Figure 2c–f). The enzymatic activities of superoxide dismutase (SOD), peroxidase (POD), catalase (CAT), and phenylalanine ammonia-lyase (PAL) were promoted after 2 h, 12 h, 2 h, and 2 h of dehydration, respectively. CAT activity reached a peak after 8 h of desiccation, while activities of the other enzymes peaked after 24 h of dehydration. This in turn lowered the O^2−^ level after 24 h of dehydration treatment in contrast to 12 h (Figure 2b), which led to dynamic changes in ROS homeostasis. Thus, our results indicate that dehydration stress triggered oxidative stress and rapid responses in tomato leaves.

### 2.3. Transcriptome Analysis Reveals DEGs in Response to Dehydration

**Figure 3:**
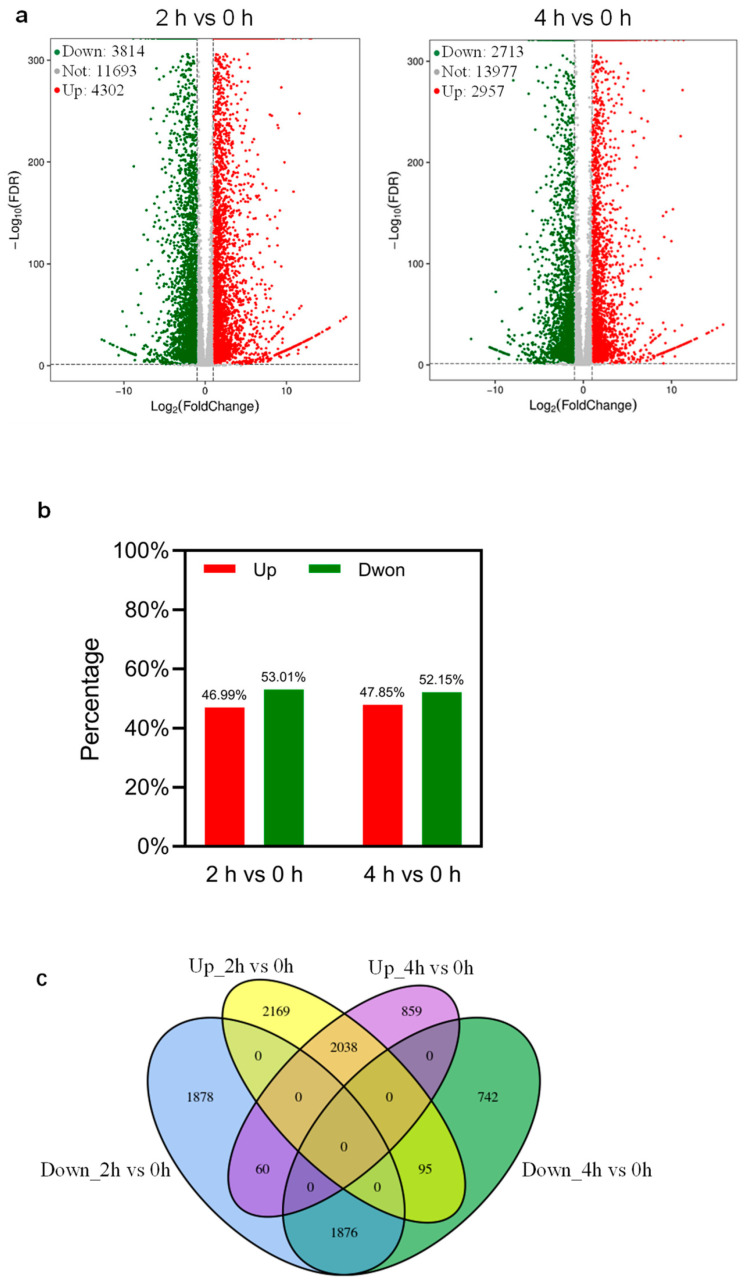
Analyses of differentially expressed genes (DEGs). (**a**) Volcano diagrams of DEGs identified in MT leaves after 2 h (left) and 4 h (right) dehydration treatment vs. 0 h. Spots above the threshold line (fold change cutoff ≥ 2, FDR < 0.05) indicate that differences are significant. Spots above the threshold line indicate that differences are significant. Downregulated genes are displayed in green, while upregulated genes are displayed in red. Genes in grey are not DEGs. (**b**) Percentage of upregulated and downregulated DEGs among total DEGs detected in 2 h and 4 h dehydration-treated MT leaves vs. 0 h. (**c**) Venn diagram representing the numbers of non-overlapped and overlapped DEGs in the four categories. Up_2 h vs. 0 h and Down_2 h vs. 0 h means upregulated and downregulated DEGs detected in 2 h dehydration-treated MT leaves vs. 0 h, respectively. Up_4 h vs. 0 h and Down_4 h vs. 0 h separately mean upregulated and downregulated DEGs detected in 4 h dehydration-treated MT leaves vs. 0 h.

In order to identify early responsive genes specifically responding to dehydration stress in tomato leaves and avoid secondary effects due to longer exposure to dehydration, the leaves of MicroTom plants treated with dehydration for 0 h, 2 h, and 4 h were profiled by RNA-seq. The expression of a considerable number of genes changed their expression when leaves were exposed to dehydration stress (Figure 3). RNA-seq analysis detected the expression of 19,809 genes in tomato leaves dehydrated for 2 h compared with 0 h control (Figure 3a). Among these genes, 8116 of them were DEGs (adjusted *p*-value cutoff <0.05 and an absolute fold change ≥2), while the other 11,693 genes were not differentially expressed. A total number of 3814 genes were downregulated and 4302 genes were upregulated after 2 h of dehydration compared with 0 h, (Figure 3a). Accordingly, 46.99% of all DEGs identified after 2 h of dehydration in contrast to 0 h were upregulated genes, and the rest, 53.01%, were downregulated genes (Figure 3b). Likewise, by comparing samples dehydrated for 4 h with 0 h control, we identified 2713 downregulated genes and 2957 upregulated genes (Figure 3a). The percentage of genes that showed repression was 47.85%, and downregulated genes made up 52.15% of total DEGs (Figure 3b). A total number of 13,977 genes were not differentially expressed in this comparison (Figure 3a). These results indicate that dehydration represses the expression of more genes.

Next, we compared the DEGs between 2 h and 4 h of dehydration-treated samples using 0 h as a control. As shown in Figure 3c, 1876 genes were commonly downregulated and 2038 commonly upregulated genes in two comparisons: 2 h vs. 0 h and 4 h vs. 0 h. Furthermore, we identified 60 genes that were downregulated after 2 h of dehydration but upregulated after 4 h of dehydration, as well as 95 genes that were upregulated first after dehydration for 2 h and then downregulated later after 4 h of dehydration (Figure 3c). In contrast to 0 h control, we also identified numerous unique DEGs including 1878 genes that were only downregulated after 2 h of dehydration, 742 genes that were only downregulated after 4 h of dehydration, 2169 genes that were only upregulated after 2 h of dehydration, and 859 genes that were only upregulated after 4 h of dehydration (Figure 3c). Together, our results suggest that the dehydration stress response at the transcriptional level is a dynamic process as changes in the expression level of different genes occurred at different time points and the duration of transcriptional changes differed among DEGs.

### 2.4. GO Analysis Reveals Genes Involved in Translation and Stress Response

**Figure 4:**
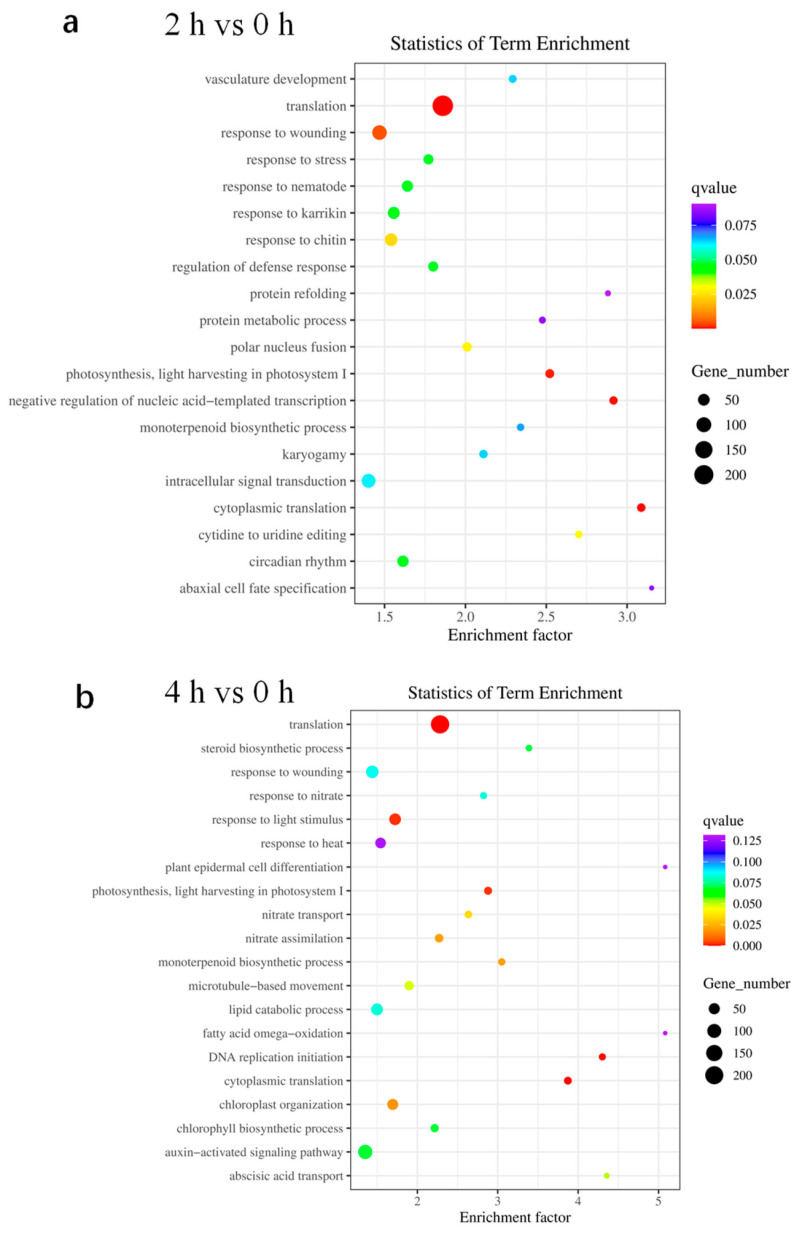
Molecular function and pathway enrichment analysis of DEGs. Gene ontology (GO) terms enrichment analysis for DEGs found in MT leaves after 2 h (**a**) and 4 h (**b**) dehydration treatment vs. 0 h. Circle sizes represent the number of genes included in the GO term while the color indicates the *q* value for the enrichment.

To illustrate functional differences of dehydration-responsive genes in tomato, GO enrichment analysis of DEGs were performed to explore significant relevant biological functions (*q* value < 0.05). In contrast to the control, many DEGs identified in leaves dehydrated for 2 h were found to be involved in translation, response to wounding, nematode, karrikin and chitin, regulation of defense response, polar nucleus fusion, photosynthesis, transcription, and cytoplasmic translation (Figure 4a). Among the DEGs found in samples dehydrated for 4 h, the most enriched categories were those involved in the translation, response to light stimulus, photosynthesis, nitrate transport and assimilation, monoterpenoid biosynthetic process, DNA replication initiation, cytoplasmic translation, and chloroplast organization (Figure 4b). Enriched GO terms from the biological processes categories were commonly found in both 2 h of dehydration vs. 0 h and 4 h of dehydration vs. 0 h data sets, including translation, photosynthesis, and cytoplasmic translation (Figure 4). The GO analysis showed that translation was the top enriched molecular function term in all groups. Taken together, the annotation of DEGs revealed that dehydration led to transcriptional responses of many translation and stress-related genes.

### 2.5. TF Families Respond to Dehydration Differentially

**Figure 5:**
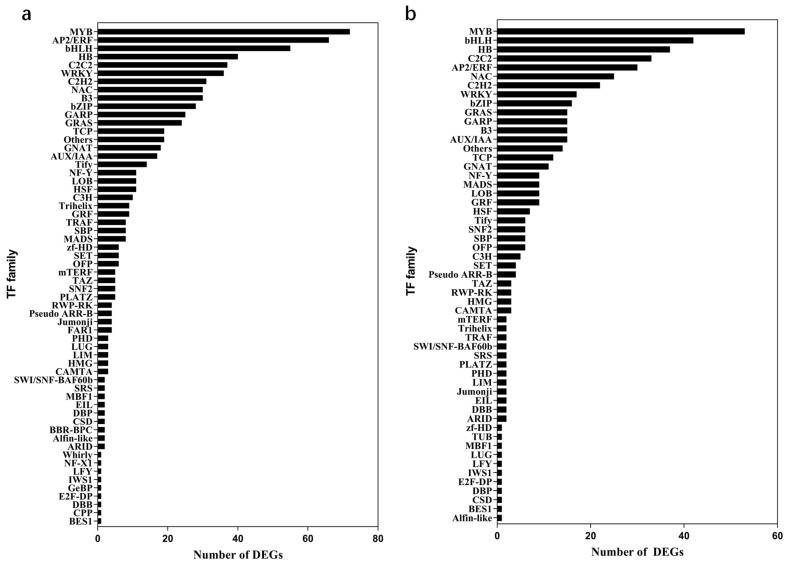
Numbers of DEGs in each transcription factor family detected in MT leaves after 2 h (**a**) or 4 h (**b**) dehydration vs. 0 h. Fold change cutoff ≥ 2 and FDR < 0.05 were used to identify differentially expressed genes.

TFs are essential regulators of drought and dehydration responses and valuable genetic resources for breeding dehydration/drought-tolerant crops [20,37,38]. To expand our knowledge of TF-mediated dehydration response, we analyzed the DEGs of all TF families. As shown in Figure 5a and Table S1a, among all the DEGs identified at the earlier time point, that is, by comparing 2 h of dehydration with 0 h control, 742 of them were annotated as TFs. Whereas, only 499 TFs were deferentially expressed in the comparison of 4 h of dehydration vs. 0 h control (Figure 5b and Table S1b).

The majority of those TF families showed responses to dehydration at both time points. A total number of 399 TFs were differentially expressed in tomato leaves at both time points, 2 h and 4 h of dehydration treatment in contrast to 0 h control (Table S1c). We also identified unique TF families that only responded to one treatment time point. For example, the BBR-BPC, CPP, FAR1, GeBP, and Whirly genes were only characterized as DEGs in samples dehydrated for 2 h, while DEGs belonging to the TUB family were only detected in leaves after 4 h of dehydration compared with 0 h control (Figure 5, Table S1a,b).

Interestingly, our results showed that MYB was the largest TF family whose expression responded to dehydration after both 2 h and 4 h of dehydration compared with 0 h control. A total number of 72 and 53 DEGs were found to be MYB TFs after 2 and 4 h of dehydration in contrast to the control, respectively (Figure 5, Table S1a,b). Other TF families containing more than 20 DEGs found in samples dehydrated for 2 h were GRAS (24), GARP (25), bZIP (28), B3 (30), NAC (30), C2H2 (31), WRKY (36), C2C2 (37), HB (40), bHLH (55), and AP2/ERF (66) (Figure 5a and Table S1a). In samples dehydrated for 4 h, C2H2 (22), NAC (25), AP2/ERF (30), C2C2 (33), HB (37), and bHLH (42) were the six TF families containing more than 20 DEGs in contrast to the control (Figure 5b and Table S1b).

**Figure 6:**
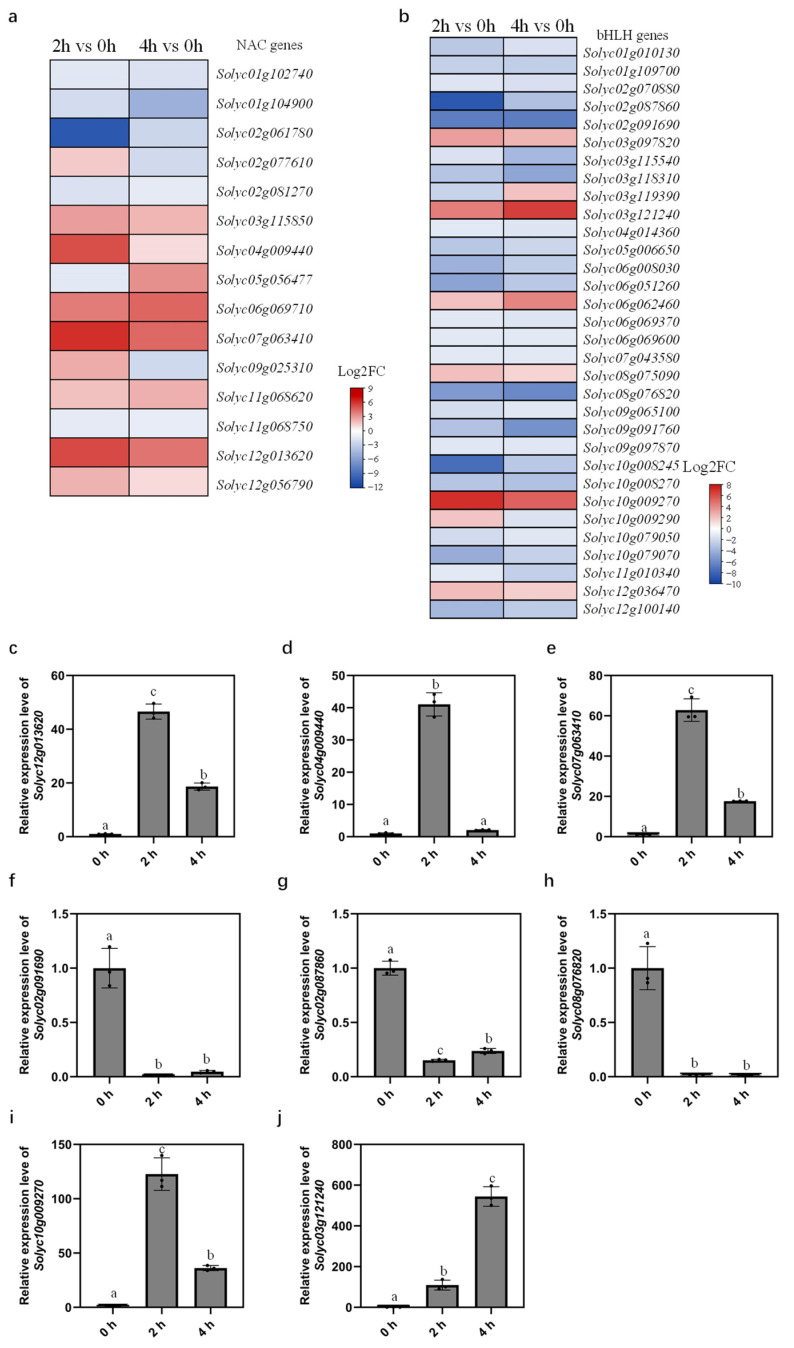
Common DEGs classified as TFs between 2 h and 4 h dehydration vs. 0 h control. (**a**,**b**) Heatmaps show transcripts abundances of differentially expressed NAC (**a**) and bHLH (**b**) TFs in 2 h and 4 h dehydration-treated MT leaves compared with 0 h. The log2 fold change (FC) scale is indicated next to the heatmap. (**c**–**j**) Relative expression levels of NAC (**c**–**e**) and bHLH (**f**–**j**) TFs measured by qRT-PCR in MT leaves after 0, 2, and 4 h dehydration treatment. In (**b**–**j**), letters indicate significant differences between means (*p* < 0.05; one-way ANOVA).

Previous studies have demonstrated that TFs of NAC, WRKY, AP2/ERF, bHLH, bZIP, HB, and WRKY families play important roles in regulating drought and dehydration responses in plants [10,32,33,39]. Here, we selected common DEGs identified in both 2 h and 4 h dehydrated samples of these TF families and showed their expressions by heatmaps. As displayed in Figure 6a and Figure S2a,b, DEGs belonging to NAC, WRKY, and bZIP families were mainly upregulated by dehydration. Out of the 15 NAC DEGs, ten of them were induced by drought after 2 h or/and 4 h of dehydration treatment compared with 0 h control (Figure 6a). The WRKY DEGs included three downregulated genes and nine upregulated genes (Figure S2a). We detected nine upregulated bZIP DEGs and four downregulated bZIP DEGs at both time points (Figure S2b). The majority of bHLH DEGs were repressed by dehydration, as 26 bHLH genes showed reduction and 8 bHLH genes showed induction of expression under dehydration stress (Figure 6b). Among the 31 HB DEGs, drought upregulated 12 of them and downregulated 20 of them (Figure S2c). The number of upregulated and downregulated DEGs in AP2/ERF and MYB families was not as distinct as the other families (Figure S2d,e).

Moreover, two AP2/ERF genes: *Solyc07g042230* (Log2FC: 11.83) and *Solyc04g071770* (Log2FC: 10.79) showed the highest induction in tomato leaves after 2 h of dehydration in contrast to 0 h control. The MYB gene *Solyc10g005550* (Log2FC:10.66) was the most upregulated TF after 4 h of dehydration, while the NAC gene *Solyc02g061780* (Log2FC: −10.32) and MYB gene *Solyc06g009480* (Log2FC: −10.62) were the most downregulated TFs in tomato leaves after 2 h and 4 h of dehydration, respectively. Therefore, our results indicate that tomato TFs of different families responded to dehydration stress in different manners: upregulation or downregulation, earlier or later response, and more or fewer changes at the transcriptional level.

### 2.6. qRT-PCR Analysis Validates Expression Patterns of DEGs

To further validate RNA-seq results, we selected a group of common DEGs annotated as TFs in leaves dehydrated for 2 h and 4 h compared with 0 h control and analyzed their expression with an alternative RNA quantification methodology (Figure 6c–j and Figure S3). As shown in Figure 6a, *Solyc12g013620*, *Solyc04g009440*, and *Solyc07g063410* were the three NAC genes whose expression exhibited the highest induction by dehydration, and the induction was higher after 2 h of dehydration treatment than 4 h. The expression levels of these NAC genes were analyzed by qRT-PCR, and they all showed the same expression patterns as the RNA-seq results (Figure 6c–e). Interestingly, Solyc04g009440, also known as SlNAC1, was previously reported to enhance tomato leaf curl virus replication, and the *SlNAC1* expression was significantly induced during *Pseudomonas* infection [40]. Hence, our study discovered new functions of Solyc04g009440 in abiotic stress in addition to its role in biotic stress.

As mentioned in the previous section, MYB is the largest family containing the most DEGs responding to dehydration stress in our study. Here, we performed qRT-PCR to analyze the expression of ten MYB genes (Figure S3f–o). Our analysis confirmed that dehydration treatment positively regulated the expression of five MYB genes (*Solyc08g082890*, *Solyc02g079280*, *Solyc06g083900*, *Solyc10g005550*, and *Solyc05g053150*) and negatively regulated the other five MYB genes (*Solyc10g076820*, *Solyc06g009480*, *Solyc02g087960*, *Solyc06g009710*, and *Solyc12g049350*). Notably, *Solyc12g049350* (*MYB11*) expression was previously reported by Zhao et al. to respond to ABA treatment [41], which is related to drought stress.

We also selected several bHLH, bZIP, WRKY, AP2/ERF, and HB genes to validate the RNA-seq data by qRT-PCR analysis. It is worth noting that these genes have not been functionally characterized so far. The qRT-PCR results verified that three bHLH genes (*Solyc02g091690*, *Solyc02g087860*, and *Solyc08g076820*) were repressed by dehydration stress, while the expression of the other two genes (*Solyc10g009270* and *Solyc03g121240*) were enhanced by dehydration (Figure 6b,f–j). We observed high consistency of RNA-seq and qRT-PCR data, as they both showed that *Solyc10g009270* was more induced after 2 h of dehydration than 4 h in contrast to 0 h control, while the expression level of *Solyc03g121240* was more induced after 4 h of dehydration (Figure 6i,j). Moreover, three WRKY genes (*Solyc02g071130*, *Solyc03g116890*, and *Solyc08g067360*), three DEGs of the HB family (*Solyc08g083130*, *Solyc01g096320*, and *Solyc02g063520*), two bZIP genes (*Solyc01g100460* and *Solyc05g050220*), and five AP2/ERF genes (*Solyc03g093610*, *Solyc12g056590*, *Solyc09g089930*, *Solyc04g071770*, and *Solyc07g042230*) all showed increased expression levels after 2 h and 4 h of dehydration treatment compared with 0 h control (Figures S2a–c and S3a–e,p–t).

By performing qRT-PCR analysis of 31 selected DEGs, we verified their expression patterns upon dehydration treatment, which was consistent with the transcriptomic data (Figure 6c–j and Figure S3). Taken together, these results indicate that those DEGs identified by RNA-seq are reliable candidate genes responding to dehydration stress, which will extend our understanding of the functions of known TFs and discovers novel tomato TFs involved in dehydration response.

## 3. Discussion

Plants have evolved different strategies to survive under drought conditions, that is, to tolerate, avoid, or escape drought stress [12]. Dehydration tolerance is an important component of drought tolerance, which is a complex trait controlled by multiple genes and largely influenced by environmental factors [3,12]. Due to climate change, there is an increasingly higher demand for dehydration/drought-tolerant varieties [42]. TFs act as regulators involved in virtually all aspects of the plant life cycle including germination, growth, development, and stress response [43,44]. They also mediate dehydration/drought response via direct or indirect regulations of ABA signaling, stomata closure, ROS, antioxidants, metabolites, and other dehydration/drought-responsive pathways [2,5,20]. These TFs can be potentially used for breeding drought-tolerant tomatoes when genetically modified properly. However, only a few tomato TFs that regulate dehydration/drought response and/or tolerance have been characterized to date. In this study, we performed RNA-seq analysis and selected 31 differentially expressed TFs of NAC, AP2/ERF, MYB, bHLH, bZIP, WRKY, and HB families in tomato leaves under dehydration stress in contrast to control. Consistently, their expression patterns were validated by qRT-PCR.

### 3.1. Comparison of the Effects of Dehydration and Drought Stress on Tomato Leaves

The relative water content is a widely used parameter in dehydration and drought-related research, which indicates the severity of stress perceived by plants. We revealed that the relative water content of tomato leaves was significantly lower after dehydration (Figure 1a,b). In accordance with this result, the MDA content and ion leakage ratio are parameters associated with membrane damage, and they showed increases after 12 h and 4 h of dehydration, respectively. These results demonstrate the similar physiological and phenotypical effects of dehydration treatment and drought stress on tomato leaves, which is in line with other studies of drought stress [9,10].

Previous studies have shown that drought stress affected the accumulation of chlorophyll in plants [45,46,47]. However, we did not observe any significant changes in chlorophyll content through the time window analyzed in this study during dehydration treatment (Figure S1). In contrast, Sakya et al. showed that drought stress decreased the total chlorophyll content of tomato leaves after several days grown under drought conditions compared with control [47], which was much longer than the drought stress we applied to tomato leaves. This might partially explain why no significant differences were detected in our experiment.

### 3.2. Dynamic Changes of ROS Levels under Dehydration Conditions

Oxidative stress caused by ROS burst is generally induced by various stresses (Zhou et al. 2019). As previously reported, drought stress affected ROS homeostasis by triggering ROS bursts and changing the activities of antioxidant enzymes in plants [11]. In this study, we detected a significant increase in H_2_O_2_ and O^2−^ content in tomato leaves after dehydration compared with the non-treated control (Figure 2a,b). This observation fully aligns with previous reports, in which we demonstrated the induction of ROS levels in tomato leaves by drought stress [48]. Moreover, activities of antioxidant enzymes including SOD, POD, CAT, and PAL were enhanced under dehydration stress and we detected the highest activities of all these enzymes after 24 h of dehydration treatment in contrast to 0 h control (Figure 2c–f), thus favoring the reduction in O^2−^ level after 24 h of dehydration compared with the earlier time point (12 h of dehydration). PAL produces phenolic compounds which capture ROS [49,50]. Although the antioxidant properties of PAL have been well elucidated in previous studies, whether it plays a role in responding to dehydration stress remains unclear. Here, we observed that the activity of PAL in tomato leaves was enhanced under dehydration conditions, suggesting the involvement of PAL in scavenging ROS under dehydration stress in tomatoes. Our results thereby point to the possibility that dehydration and drought stress induce common responsive pathways in tomato leaves.

### 3.3. Transcriptome Profiling Identifies Dehydration-Responsive Genes

In order to unravel the molecular mechanism of dehydration response in tomatoes at the transcriptional level, we conducted RNA-seq analysis using tomato leaves treated with or without dehydration and compared the transcriptome of samples after 2 or 4 h of dehydration with 0 h control. Genes that significantly changed in expression due to dehydration treatment by at least 2-fold (up or down) with adjusted *p*-value < 0.05 were considered as DEGs. The RNA-seq data revealed 8116 DEGs after 2 h of dehydration and 5670 DEGs after 4 h of dehydration compared with the control (Figure 3a,b). GO analysis indicated that these DEGs were mainly involved in translation, photosynthesis, stress response, and cytoplasmic translation (Figure 4). All these pathways are indeed related to stress response or adaptation to environmental stimuli in plants. Thus, our results identified novel candidate genes in tomatoes involved in the regulation of dehydration response. An interesting question that remains to be addressed is whether the involvement of these DEGs in dehydration and drought response can be experimentally verified. Addressing this question in detail requires the analysis of loss and gain function mutants of these TFs, which will be an important task in the future.

### 3.4. Differentially Expressed TFs

TFs are valuable genetic resources for breeding dehydrated/drought-tolerant varieties. A total number of 742 TFs belonging to MYB, AP2/ERF, bHLH, HB, and other families were differentially expressed in samples dehydrated for 2 h compared with 0 h control (Figure 5a). By contrast, we only identified 499 DEGs annotated as TFs in tomato leaves after 4 h of dehydration (Figure 5b), suggesting that some TFs responded to dehydration stress and adjusted their expression back to normal levels rapidly. Notably, TFs showing earlier response to dehydration may act more upstream of the signal transduction pathway. The majority of TFs that responded to dehydration stress identified by RNA-seq belonged to MYB, GRAS, GARP, bZIP, B3, NAC, C2H2, WRKY, C2C2, HB, bHLH, and AP2/ERF family (Figure 5, Table S1). Some TF families, for example NAC and WRKY, have been previously reported to regulate dehydration response [10,33,34]. However, whether TFs of C2H2, C2C2, GARP, and B3 families play roles in the response to dehydration stress has not been reported.

As shown in Figure 6c–j and Figure S3, we performed qRT-PCR analysis to examine the expression levels of 31 differentially expressed TFs identified by RNA-Seq and confirmed the upregulation or downregulation of these TFs by dehydration stress. The same results obtained by the two methods suggest that these differentially expressed TFs were indeed dehydration-responsive genes. Notably, the involvement of these differentially expressed TFs in dehydration or drought response has not yet been reported. Our study thus provides illuminating insights into the regulatory networks of dehydration response in tomatoes.

Moreover, we compared the expression levels of six drought-responsive marker genes: *SlDREB1* (*Solyc06g050520*), *SlDREB2* (*Solyc12g008350*), *SlDREB3* (*Solyc04g072900*), SlGRAS4 (*Solyc01g100200*), SlNCED (*Solyc07g056570*), and *SlGA2OX7* (*Solyc02g080120*), which were previously reported to positively respond to drought stress [51,52,53,54]. Our results showed that these genes were upregulated by dehydration treatment, suggesting the identification of overlapping regulators of drought and dehydration response by transcriptomic profiling in this study (Figure S4).

Given the overlapping responsive genes in tomato leaves under drought and dehydration stress, those dehydration-responsive TFs identified in this study are likely to control drought response pathways as well. Thus, they are promising targets to improve the dehydration/drought tolerance of tomatoes for breeders. However, functional analysis of the differentially expressed TFs; for instance, their expression patterns under dehydration and drought stress conditions in different tissues, phenotypes of their knockout mutants and overexpressors, and the regulatory networks controlled by them remain to be elucidated. These will be key aspects of future research to discover new strategies for improving the dehydration/drought tolerance of tomato cultivars.

## 4. Methods and Plant Materials

### 4.1. Plant Material and Growth Conditions

*Solanum lycopersicum* L. cv. MicroTom (MT) was used in this study. Tomato seeds preserved in our laboratory were sown in commercially available tomato-cultivation soil and grown in a chamber at 25 °C, 250 μmol m^−2^ s^−1^ photos irradiance, with a relative humidity of 75%, under a light/darkness cycle of 16/8 h regulated by fluorescent lamps. Tomato plants were watered with a nutrient solution once a week. For desiccation, the 4th leaves of one-month-old MT plants were detached at 8 am and placed on filter papers for air-drying in the growth chamber for 0–24 h as described previously [10].

### 4.2. Determination of Relative Water Content

The relative water content (RWC) of leaves was determined as described previously [10]. Briefly, detached leaves were weighed immediately to determine the fresh weight (FW). Subsequently, leaves were immersed in distilled water and incubated at 4 °C overnight to obtain the saturated weight (SW). Leaves were then dried at 60 °C for 24 h to measure the dry weight (DW). RWC was calculated using the formula RWC% = (FW − DW)/(SW − DW) × 100%.

### 4.3. Determination of Ion Leakage

Leaves were immersed in 50 mL of deionized water and shaken at room temperature for 12 h. Initial electrical conductivity was measured at 25 °C using a conduct meter (INESA, Shanghai, China). Later, samples were boiled at 100 °C for 30 min and cooled down at room temperature until 25 °C, and total conductivity was measured again. Ion leakage is shown as the percentage of initial conductivity of the total conductivity; low and high percentage values indicate less or more membrane damage, respectively [9].

### 4.4. Enzyme Measurements

Measurements of enzyme activities were performed as described previously [10]. Samples were ground to a fine powder in liquid nitrogen and 100 mg powder was homogenized in 500 μL lysis buffer containing 50 mM Tris–HCl (pH 7.8), 0.1 mM EDTA, 0.1% (*w*/*v*) Triton X-100 and 1% (*w*/*v*) polyvinylpolypyrrolidone (PVPP). Samples were centrifuged at 10,000× *g* for 10 min, and supernatants were used for further measurements. Assay kits of POD, CAT, SOD, and PAL (JC DETECT, Nanjing, China) were used for the determination of their activities according to the respective manual. The absorbance of reaction mixtures was measured by Infinite 200 Pro M Nano plate reader (TECAN, Männedorf, Switzerland).

### 4.5. Quantification of H_2_O_2_, O^2−^, and Malondialdehyde (MDA)

Lipid peroxidation was assessed by measuring MDA levels with the MDA assay kit (JC DETECT, Nanjing, China). For the quantification of H_2_O_2_ and O^2−^ content, assay kits (JC DETECT, Nanjing, China) were used. The absorbance of reaction mixtures was measured by Infinite 200 Pro M Nano plate reader (TECAN, Männedorf, Switzerland) according to manuals of MDA, H_2_O_2_, and O^2−^ assay kit.

### 4.6. Total RNA Extraction

The 4th leaves of one-month-old MT plants were harvested after 0, 2, and 4 h of dehydration treatment and frozen immediately in liquid nitrogen. The total RNA was extracted from MT leaves as described previously [55,56]. Briefly, samples were ground into fine powder for RNA extraction using RNAprep Pure Plant Plus kit (TIANGEN, Beijing, China). RNA degradation and contamination were monitored on 1% agarose gels. RNA purity and concentration were measured using the NanoPhotometer^®^ spectrophotometer (IMPLEN, Westlake Village, CA, USA). RNA integrity was assessed using the RNA Nano 6000 Assay Kit of the Agilent Bioanalyzer 2100 system (Agilent Technologies, Santa Clara, CA, USA).

### 4.7. Library Construction and RNA-Seq Data Analysis

Library preparation and sequencing were performed at Genepioneer, Nanjing, China (http://www.genepioneer.com/ (accessed on 29 May 2023)). RNA-seq was performed with three biological replicates per sample on the Nova 6000 platform (Illumina, San Diego, CA, USA). Sequencing adaptors and low-quality reads were trimmed using Trimmomatic [57]. Paired-end clean reads were then aligned to the tomato reference genome SL4.0 using HISAT2 [58]. FPKM of each gene was calculated by StringTie based on the length of the gene and the read count mapped to this gene [59]. Differential expression analysis was performed using the DESeq R package [60]. An adjusted *p*-value cutoff <0.05 and an absolute fold change ≥2 were used to identify differentially expressed genes. The RNA-seq data are available from the NCBI BioProject database (www.ncbi.nlm.nih.gov/bioproject (accessed on 29 May 2023)) under ID PRJNA947261.

### 4.8. Gene Ontology (GO) Enrichment Analysis of Differentially Expressed Genes (DEGs)

Gene function was annotated based on the Gene Ontology database as described previously. GO enrichment analysis of the DEGs was implemented by the GOseq R packages based on Wallenius non-central hyper-geometric distribution [61].

### 4.9. Quantitative Real-Time PCR (qRT-PCR)

cDNA synthesis and qRT-PCR were performed as described previously (Dong et al. 2022). Primers were designed using QuantPrime [62]. Oligonucleotides were obtained from Tsingke, Beijing, China (https://tsingke.com.cn/ (accessed on 29 May 2023)). Primer sequences are given in Table S2. PCR reactions were run on a QuantStudio™ 6 Flex sequence detection system (Applied Biosystems, Foster City, CA, USA), and amplification products were visualized using SYBR Green (Life Technologies, Carlsbad, CA, USA) with *SlGAPDH* (*Solyc04g009030*) as the reference gene.

### 4.10. Data Presentation and Statistical Analysis

The software GraphPad Prism 9 (GraphPad Software Inc., San Diego, CA, USA) was used for plotting the experimental data and analyzing significant levels of differences with one-way ANOVA at *p* < 0.05.

## 5. Conclusions

In this study, we investigated the phenotypical and physiological changes of tomato leaves after 0, 2, 4, 6, 8, 12, and 24 h of dehydration treatment. Our results showed that the most severe damages caused by dehydration occurred after 24 h of dehydration. Similar to drought stress, dehydration treatment enhanced the accumulation of ROS and the activities of antioxidant enzymes. The transcriptomic profiling of tomato leaves after 0, 2, and 4 h of dehydration identified a considerable number of DEGs including TFs of different families and drought-responsive genes. The expression patterns of 31 differentially expressed TFs were verified by qRT-PCR. Taken together, the novel dehydration-responsive TFs identified in this study might be promising targets for breeding dehydration/drought-tolerant tomato varieties in the future.

## Data Availability

The raw sequencing data of this article are stored in the NCBI Sequence Read Archive under accession number PRJNA947261.

## Supplementary Materials

The supporting information can be downloaded at: https://www.mdpi.com/article/10.3390/ijms24119725/s1.
