# Supplementary material for: Transcriptomic Profiling of Tomato Leaves Identifies Novel Transcription Factors Responding to Dehydration Stress
Source: Int J Mol Sci. 2023 Jun 3;24(11):9725. doi: 10.3390/ijms24119725 (PMC10253658; doi:10.3390/ijms24119725)
Supplement: Supplementary file 1 [file ijms-24-09725-s001.zip › Supplemental Figures-2023 June-revised.pdf]

Transcriptomic profiling identifies novel transcription factors involving drought response in tomato

Shuchao Dong[1,2], Jiayi Lin[1,3], Liuxia Song[1,2], Liping Zhao[1,2], Yinlei Wang[1,2], and Tongmin Zhao[1,2§]

[1]Institute of Vegetable Crop，Jiangsu Academy of Agricultural Sciences, Nanjing 210014, China;

[2]Jiangsu Key Laboratory for Horticultural Crop Genetic Improvement，Nanjing 210014，China; [3]College of Horticulture and Plant Protection, Yangzhou University, Yangzhou 225100, China

[§]Corresponding author: Tongmin Zhao: tmzhaomail@163.com

Figure S1

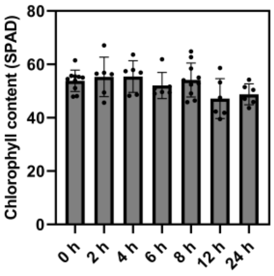

Figure S2

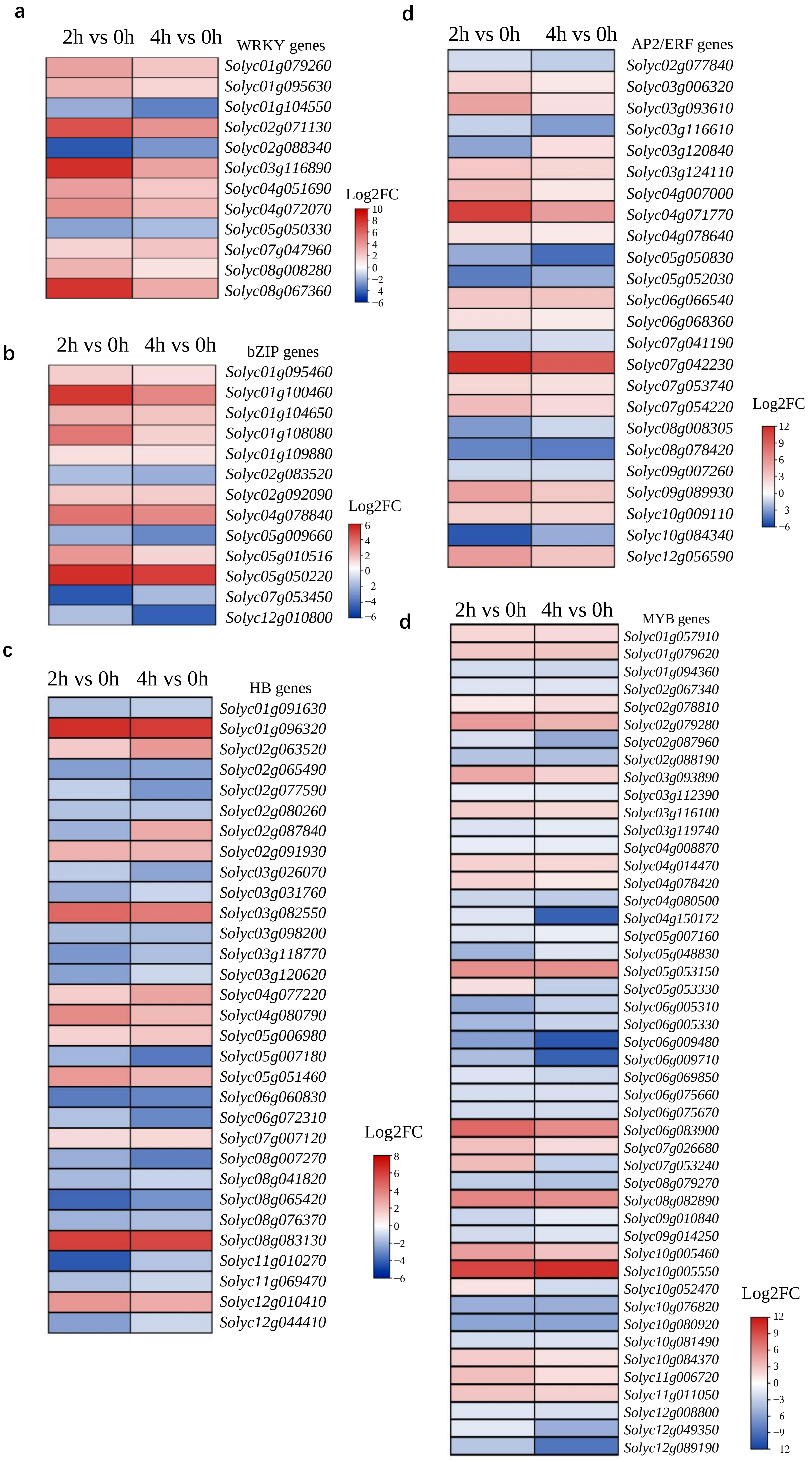
a
2h vs 0h
4h vs 0h
WRKY genes
Solyc01g079260
Solyc01g095630
Solyc01g104550
Solyc02g071130
Solyc02g088340
Solyc03g116890
Solyc04g051690
Solyc04g072070
Solyc05g050330
Solyc07g047960
Solyc08g008280
Solyc08g067360
Log2FC
10
8
6
4
2
0
−2
−4
−6
b
2h vs 0h
4h vs 0h
bZIP genes
Solyc01g095460
Solyc01g100460
Solyc01g104650
Solyc01g108080
Solyc01g109880
Solyc02g083520
Solyc02g092090
Solyc04g078840
Solyc05g009660
Solyc05g010516
Solyc05g050220
Solyc07g053450
Solyc12g010800
Log2FC
6
4
2
0
−2
−4
−6
c
2h vs 0h
4h vs 0h
HB genes
Solyc01g091630
Solyc01g096320
Solyc02g063520
Solyc02g065490
Solyc02g077590
Solyc02g080260
Solyc02g087840
Solyc02g091930
Solyc03g026070
Solyc03g031760
Solyc03g082550
Solyc03g098200
Solyc03g118770
Solyc03g120620
Solyc04g077220
Solyc04g080790
Solyc05g006980
Solyc05g007180
Solyc05g051460
Solyc06g060830
Solyc06g072310
Solyc07g007120
Solyc08g007270
Solyc08g041820
Solyc08g065420
Solyc08g076370
Solyc08g083130
Solyc11g010270
Solyc11g069470
Solyc12g010410
Solyc12g044410
Log2FC
8
6
4
2
0
−2
−4
−6
d
2h vs 0h
4h vs 0h
AP2/ERF genes
Solyc02g077840
Solyc03g006320
Solyc03g093610
Solyc03g116610
Solyc03g120840
Solyc03g124110
Solyc04g007000
Solyc04g071770
Solyc04g078640
Solyc05g050830
Solyc05g052030
Solyc06g066540
Solyc06g068360
Solyc07g041190
Solyc07g042230
Solyc07g053740
Solyc07g054220
Solyc08g008305
Solyc08g078420
Solyc09g007260
Solyc09g089930
Solyc10g009110
Solyc10g084340
Solyc12g056590
Log2FC
12
9
6
3
0
−3
−6
d
2h vs 0h
4h vs 0h
MYB genes
Solyc01g057910
Solyc01g079620
Solyc01g094360
Solyc02g067340
Solyc02g078810
Solyc02g079280
Solyc02g087960
Solyc02g088190
Solyc03g093890
Solyc03g112390
Solyc03g116100
Solyc03g119740
Solyc04g008870
Solyc04g014470
Solyc04g078420
Solyc04g080500
Solyc04g150172
Solyc05g007160
Solyc05g048830
Solyc05g053150
Solyc05g053330
Solyc06g005310
Solyc06g005330
Solyc06g009480
Solyc06g009710
Solyc06g069850
Solyc06g075660
Solyc06g075670
Solyc06g083900
Solyc07g026680
Solyc07g053240
Solyc08g079270
Solyc08g082890
Solyc09g010840
Solyc09g014250
Solyc10g005460
Solyc10g005550
Solyc10g052470
Solyc10g076820
Solyc10g080920
Solyc10g081490
Solyc10g084370
Solyc11g006720
Solyc11g011050
Solyc12g008800
Solyc12g049350
Solyc12g089190
Log2FC
12
9
6
3
0
−3
−6
−9
−12

Figure S3

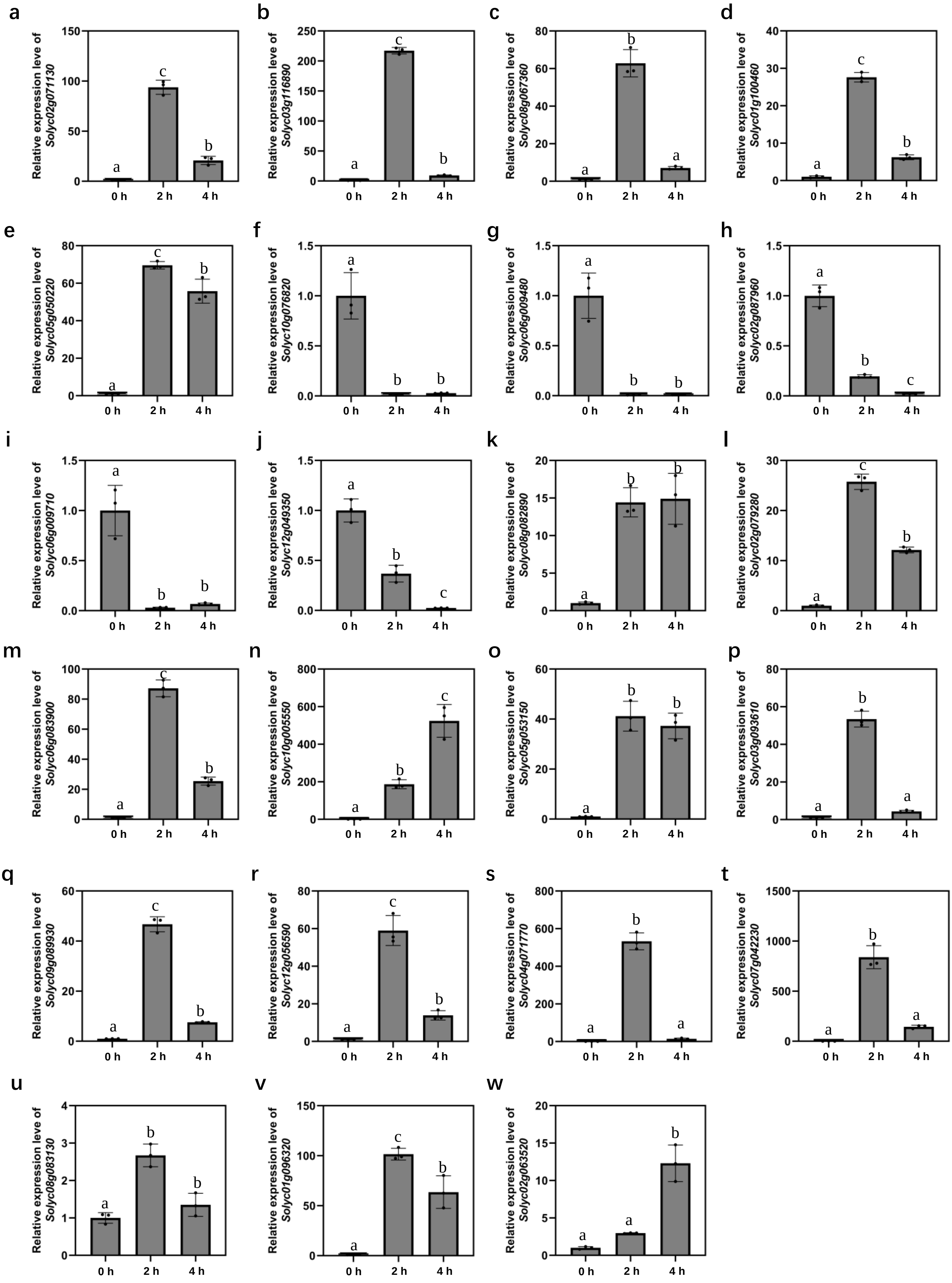
a
Relative expression leve of Solyc02g071130
b
Relative expression leve of Solyc03g116890
c
Relative expression leve of Solyc08g067360
d
Relative expression leve of Solyc01g100460
e
Relative expression leve of Solyc05g050220
f
Relative expression leve of Solyc10g076820
g
Relative expression leve of Solyc06g009480
h
Relative expression leve of Solyc02g087960
i
Relative expression leve of Solyc06g009710
j
Relative expression leve of Solyc12g049350
k
Relative expression leve of Solyc08g082890
l
Relative expression leve of Solyc02g079280
m
Relative expression leve of Solyc06g083900
n
Relative expression leve of Solyc10g005550
o
Relative expression leve of Solyc05g053150
p
Relative expression leve of Solyc03g093610
q
Relative expression leve of Solyc09g089930
r
Relative expression leve of Solyc12g056590
s
Relative expression leve of Solyc04g071770
t
Relative expression leve of Solyc07g042230
u
Relative expression leve of Solyc08g083130
v
Relative expression leve of Solyc01g096320
w
Relative expression leve of Solyc02g063520
0 h
2 h
4 h

Figure S4

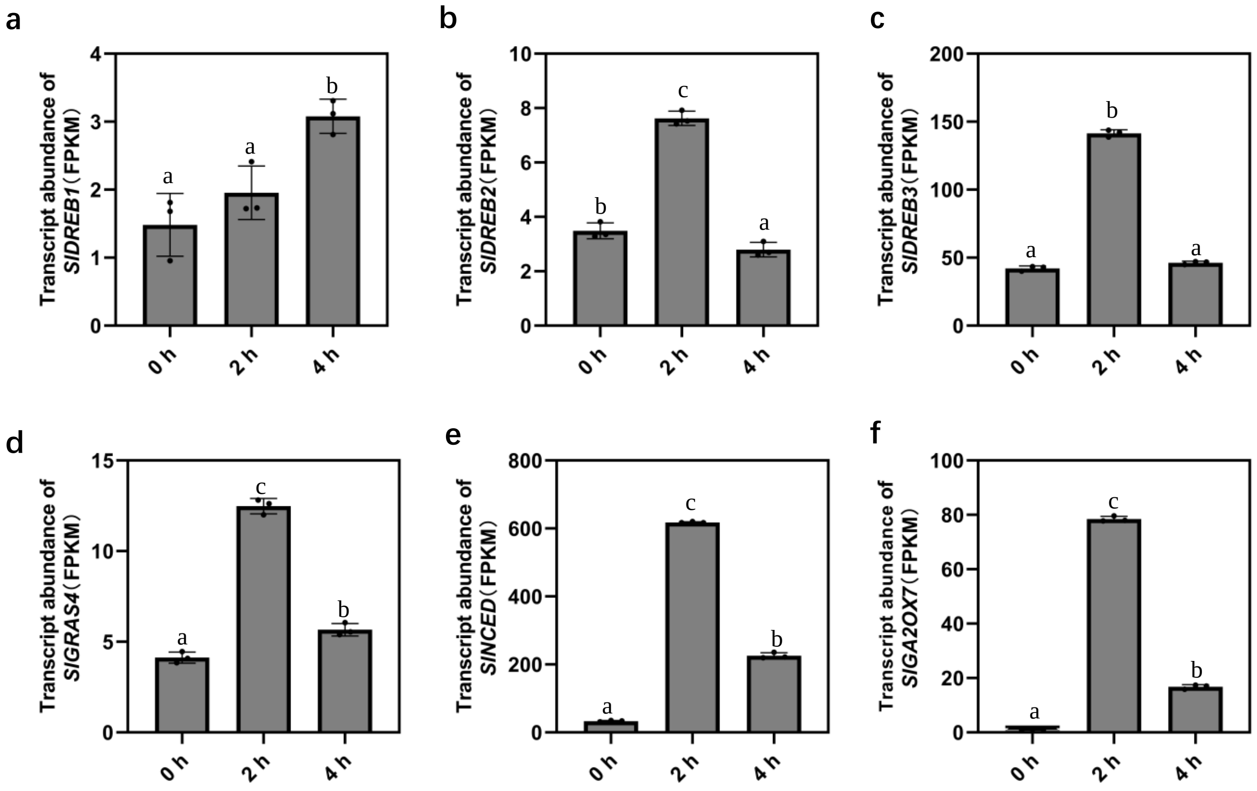
a
b
c
d
e
f
Transcript abundance of SlDREB1 (FPKM)
Transcript abundance of SlDREB2 (FPKM)
Transcript abundance of SlDREB3 (FPKM)
Transcript abundance of SlGRAS4 (FPKM)
Transcript abundance of SlNCED (FPKM)
Transcript abundance of SlGA2OX7 (FPKM)
0 h
2 h
4 h
